# Three Decades of Monitoring Reveal That Differing Population Trends of Large Herbivores in Gonarezhou National Park, Zimbabwe, Are Related to Species Body Mass

**DOI:** 10.1002/ece3.74110

**Published:** 2026-08-13

**Authors:** Kevin M. Dunham

**Affiliations:** ^1^ Independent Researcher Harare Zimbabwe

**Keywords:** aerial surveys, African savannah elephant, elephant impacts, predation, rainfall

## Abstract

Trends in large herbivore abundance in Gonarezhou National Park, Zimbabwe, were examined using population estimates from aerial surveys designed for elephant *Loxodonta africana* . For ~20 years after the particularly severe 1992 drought, the numbers of elephant, buffalo *Syncerus caffer* , eland *Tragelaphus oryx*, giraffe *Giraffa camelopardalis* , greater kudu *Tragelaphus strepsiceros* , nyala *Tragelaphus angasii* , warthog *Phacochoerus africanus* , waterbuck *Kobus ellipsiprymnus* , wildebeest *Connochaetes taurinus* and zebra *Equus quagga* increased. After 2013/2014, survey estimates for buffalo, eland, elephant, giraffe and zebra increased more slowly, suggesting that a logistic curve was an appropriate model for these species. In contrast, estimates for impala, kudu, nyala, warthog and wildebeest decreased after 2013. Species that declined had a body mass < 140 kg and species that did not had a mass ≥ 200 kg, which suggests that predation likely caused the declines. It is less obvious why trends in the abundance of smaller herbivores changed from increasing before 2013 to decreasing afterwards. Survey estimates for elephants in female herds suggest that there was an abrupt reduction in their rate of increase between the period before 2014 and that afterwards. Most herbivore population declines occurred during that latter period, when the number of elephants in female herds essentially stabilised, likely as a result of food limitation. From studies elsewhere, one can hypothesise that in Gonarezhou: (1) there was direct competition for food between elephant and the browser and mixed feeder species; (2) the food supply for grazers was reduced by dry‐season rainfall often being below‐average; (3) food shortages lead to poorer body condition and increased vulnerability to predation; and (4) elephant impacts on woody vegetation structure further increased the vulnerability of smaller herbivores to predation. The numerous elephants in Gonarezhou might appear a conservation success, but their high density may have adversely impacted populations of some large herbivores and predators.

## Graphical abstract

For ~20 years after the severe 1992 drought, the estimated numbers of elephant, buffalo, eland, giraffe, greater kudu, nyala, warthog, waterbuck, wildebeest and zebra increased in Gonarezhou NP, Zimbabwe. After 2013/2014, the rate of change in the survey estimates declined for buffalo, eland, elephant, giraffe and zebra, but was not negative, suggesting that a logistic curve was an appropriate model for these species. In contrast, estimates for impala, kudu, nyala, warthog and wildebeest decreased after 2013. Species that declined had a body mass < 140 kg and species that did not had a mass ≥ 200 kg. This paper discusses the possible roles of rainfall, predation and elephant impacts to the vegetation in causing the differing population trends.

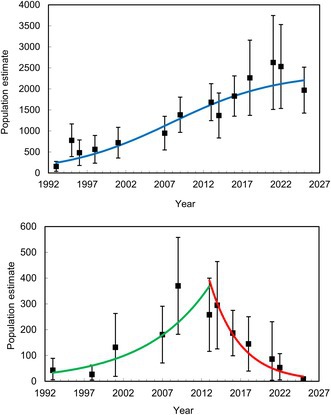

## 1 Introduction

Rainfall is an important determinant of plant biomass and thus of the food supply for and the abundance of large herbivores in African savannahs (Coe et al. 1976; East 1984; Fritz and Duncan 1994; Ogutu et al. 2008). Rainfall during the dry season can be an important determinant of the food supply for grazing ungulates during the time of the year when the availability of green grass is limited (Mduma et al. 1999; Dunham et al. 2004). But this bottom‐up process may be overridden by top‐down processes from predators (Sinclair and Krebs 2002). In Serengeti NP, Tanzania, there is a range of large mammalian predators and herbivores, diverse in terms of species and size (Sinclair et al. 2003). The main cause of mortality for adults of any particular herbivore species was determined by two factors: the species diversity of both the predators and prey; and the body size of that particular prey species, relative to predators and other herbivores. Small ungulate species were exposed to more predator species, and experienced greater predation rates, than larger ungulate species. There was a threshold at prey body sizes of ~150 kg, above which ungulate species had few natural predators. Below this threshold, prey populations were regulated by predation; while above it, prey populations exhibited food limitation. Sinclair et al. (2003) suggested that this may apply generally in systems where there is a diversity of predators and prey. The relative importance of predation, food quantity and food quality in regulating African savanna populations of large herbivores of increasing body mass is predicted to vary across rainfall and soil fertility gradients (Hopcraft et al. 2010). It is suggested that: predation regulates small herbivores when soil nutrient status is high, regardless of rainfall; that food quality regulates all herbivores (with predation acting synergistically at small size) when rainfall is high and soil nutrient status low; and that predation is not regulating when both rainfall and soil nutrient status are low. Sudden shifts in primary production caused by abiotic disturbances such as fire, or marked changes in consumption rates (herbivory, predation, or disease) may rearrange the dynamics of an ecosystem.

When at high density, elephants (scientific names in Table 1) can extensively modify woodland structure by felling or debarking trees (Thomson 1975). A comparison of wildlife densities in Eastern and Southern Africa ecosystems found that megaherbivores, primarily elephants, did not affect mesograzers (Fritz et al. 2002). But there were negative relationships between the relative abundances of elephants and mesobrowsers, and of elephants and mesomixed feeders. This suggested that elephants outcompeted the mesobrowser and mesomixed feeder species for food, or that they altered the vegetation to make it unfavourable for these species, either as food resources or as cover from predators. Increases in elephant densities in the Sabi Sands‐MalaMala complex, South Africa, and the consequent changes in tree density and the vertical availability of browse explained the population fluctuations of other herbivore species through the impact of elephants on the food supply of these other species (de Boer et al. 2015). The biomass density of grazers increased more than that of the browsers, shifting the community toward a grazer and megaherbivore‐dominated one. Mesobrowsers were unable to benefit from the impact of elephants on trees, but large grazing species responded positively. Hence, changes in elephant densities can trigger cascading effects on communities of large herbivores.

**TABLE 1:** Years for which survey estimates of large herbivore species are available for Gonarezhou NP during 1993–2025.

	Year of survey													
	1993	1995	1996	1998	2001	2007	2009	2013	2014	2016	2018	2021	2022	2025
Species														
Buffalo *Syncerus caffer* ^a^	+	+	+	+	+	+	+	+	+	+	+	+	+	+
Common Duiker *Sylvicapra grimmia*		+	+		+	+	+	+	+	+	+	+	+	+
Eland *Tragelaphus oryx* ^b^	+	+	+	+	+	+	+	+	+	+	+	+	+	+
Savannah elephant *Loxodonta africana*	+^c^	+	+	+	+	+	+	+	+	+	+	+	+	+
Giraffe *Giraffa camelopardalis*	+	+	+	+	+	+	+	+	+	+	+	+	+	+
Impala *Aepyceros melampus*		+	+	+	+	+	+	+	+	+	+	+	+	+
Kudu *Tragelaphus strepsiceros*	+	+	+	+	+	+	+	+	+	+	+	+	+	+
Nyala *Tragelaphus angasii*	+	+^d^	+^d^	+	+	+	+	+	+	+	+	+	+	+
Ostrich *Struthio camelus*	+	+	+	+	+	+	+	+	+	+	+	+^d^	+^d^	+
Steenbok *Raphicerus campestris*						+	+	+	+	+	+	+	+	+
Warthog *Phacochoerus africanus*			+		+	+	+	+	+	+	+	+	+	+
Waterbuck *Kobus ellipsiprymnus*	+	+	+	+	+	+	+	+	+	+	+	+	+	+
Wildebeest *Connochaetes taurinus* ^e^	+^d^	+	+^d^	+	+^d^	+	+	+	+	+	+	+	+	+
Zebra *Equus quagga*	+	+	+	+	+	+	+	+	+	+	+	+	+	+
Elephant social group														
Elephants in female herds		+	+	+	+	+	+	+	+	+	+	+	+	+
Elephants in bull groups		+	+	+	+	+	+	+	+	+	+	+	+	+

Abbreviation: +, survey estimate available.

^a^ ~600 buffaloes were released during 1998–2000 and so the population trend was determined for 2001–2025.

^b^ The 2013 survey occurred soon after the release of 96 elands. The survey estimates for eland often had large confidence intervals and so the impact, if any, on the population trend of this release is not obvious. The released animals were not monitored and so the impact would have been only slight if a high proportion of the freed eland died or left the park.

^c^ Some elephants were captured while the 1993 survey was underway and so the population trend was determined for 1995–2025.

^d^ Zero estimate. The estimates for nyala and wildebeest suggested that they were uncommon during the decade after the drought, but common during 2007 and 2009. The zero estimates probably resulted from sampling error, with the species so uncommon that none were seen along the transects. Hence, trends for nyala and wildebeest were determined using only the survey estimates greater than zero.

^e^ The 2013 survey occurred soon after the release of 390 wildebeests. The population trend was determined for 1995–2025, ignoring the zero estimates for 1996 and 2001, as well as the 2013 estimate, which was the peak estimate.

Elephant impacts on woodland can also influence the vulnerability of other large herbivores to predation. A study of microhabitat selection by five herbivore species (steenbok, impala, kudu, zebra and giraffe) in Hwange NP, Zimbabwe, revealed that they did not strongly and consistently avoid sites with elephant‐induced vegetation changes (Valeix et al. 2011). The results of the study did not support the hypothesis that population declines of the studied herbivores were caused by the direct effects of elephants on vegetation structure. But they did suggest that perceived predation risk influenced microhabitat selection by large herbivores, and that elephants, by modifying the visibility of habitats, may have strong, indirect effects on predator–prey relationships.

In Addo Elephant NP, South Africa, the reintroduction of lion *Panthera leo* and spotted hyaena *Crocuta crocuta* was followed by declines in the numbers of small to medium sized herbivores in an area with a high density of elephants, but not in an area where elephants had been absent for ~100 years (Tambling et al. 2013). This suggested that elephant impacts on the woody vegetation, specifically the opening up of thickets, increased the vulnerability of the herbivores to predation. In a study of lion kills and vegetation structure in Hwange, elephant‐induced vegetation changes tended to be associated with an increase in both visibility and the distance to a potential ambush site (Ferry et al. 2020). At a large scale, more lion kills were located in woody vegetation characterised by greater elephant impacts, but there was no selection for reduced visibility, or a shorter distance to a potential ambush site. Other factors, such as prey abundance, may drive lion hunting behaviour at this scale. At a microhabitat scale, lion kills were preferentially located in habitats characterised by a shorter distance to a potential ambush site. In Hluhluwe‐iMfolozi Park, South Africa, an experiment that simulated elephant‐induced habitat changes at both patch scale (removal of woody vegetation) and within‐patch scale (deposition of coarse woody debris) and then recorded use by impala and warthog suggested that the perceived landscape of fear of predators for large herbivores was both species‐specific and dynamic, varying in space and time (Fležar et al. 2019). The effect on predation vulnerability of elephant impacts to the structure of woody vegetation would likely vary between predator species, and depend on the initial vegetation structure. In Addo, the opening up of thickets by elephant facilitated access by lion, but in more open vegetation, stalkers (e.g., lion and leopard *Panthera pardus* ) would be expected to benefit from additional low woody plants and obstacles, which form potential ambush sites. Coursers (e.g., cheetah *Acinonyx jubatus* and wild dog *Lycaon pictus* ) would be expected to benefit from more open vegetation, with fewer obstacles to hamper the chase.

The first purpose of this study is to describe the trends in the abundance of large herbivores in Zimbabwe's Gonarezhou National Park (hereafter Gonarezhou) during the past three decades, assuming that data from elephant surveys provide accurate estimates of real trends in the populations of other large herbivores. Surveys conducted up until 2009 revealed the population trends for large herbivores in Gonarezhou separately for the periods before and after the particularly severe 1992 drought (Dunham 2012). The rainy season is typically November to April, but during March 1992 this drought was described as ‘the worst drought in living memory. In the south‐east lowveld [of Zimbabwe], the season's rainfall ……… has ranged from 20 mm to 100 mm. This, in conjunction with high temperatures and endless cloudless days, has meant that there has been no summer growth. In addition, there is very limited browse, and in some areas, the trees have not flushed at all. There is therefore a total lack of food’ (Tayler 1992). The impact of the drought on the Gonarezhou wildlife was not monitored other than by the aerial survey programme, but anecdotal reports suggest that there was widespread mortality and that all large herbivores were seriously impacted (Tayler 1992). Survey estimates for all large herbivore species were low immediately after the drought. The longer time series of estimates now available from surveys conducted since the drought reveals that recently population trends have differed between large herbivore species in Gonarezhou.

The second purpose of this study is to consider possible reasons for the population trends differing between species. Determining the causes of these differences is not possible retrospectively, but correlations between the population trends and environmental factors may suggest reasons. The possible roles of rainfall, predation and elephant impacts on the vegetation are considered. No single factor provides a complete explanation for the observed trends, and several factors may have acted together.

## 2 Materials and Methods

### 2.1 Study Area

**FIGURE 1:**
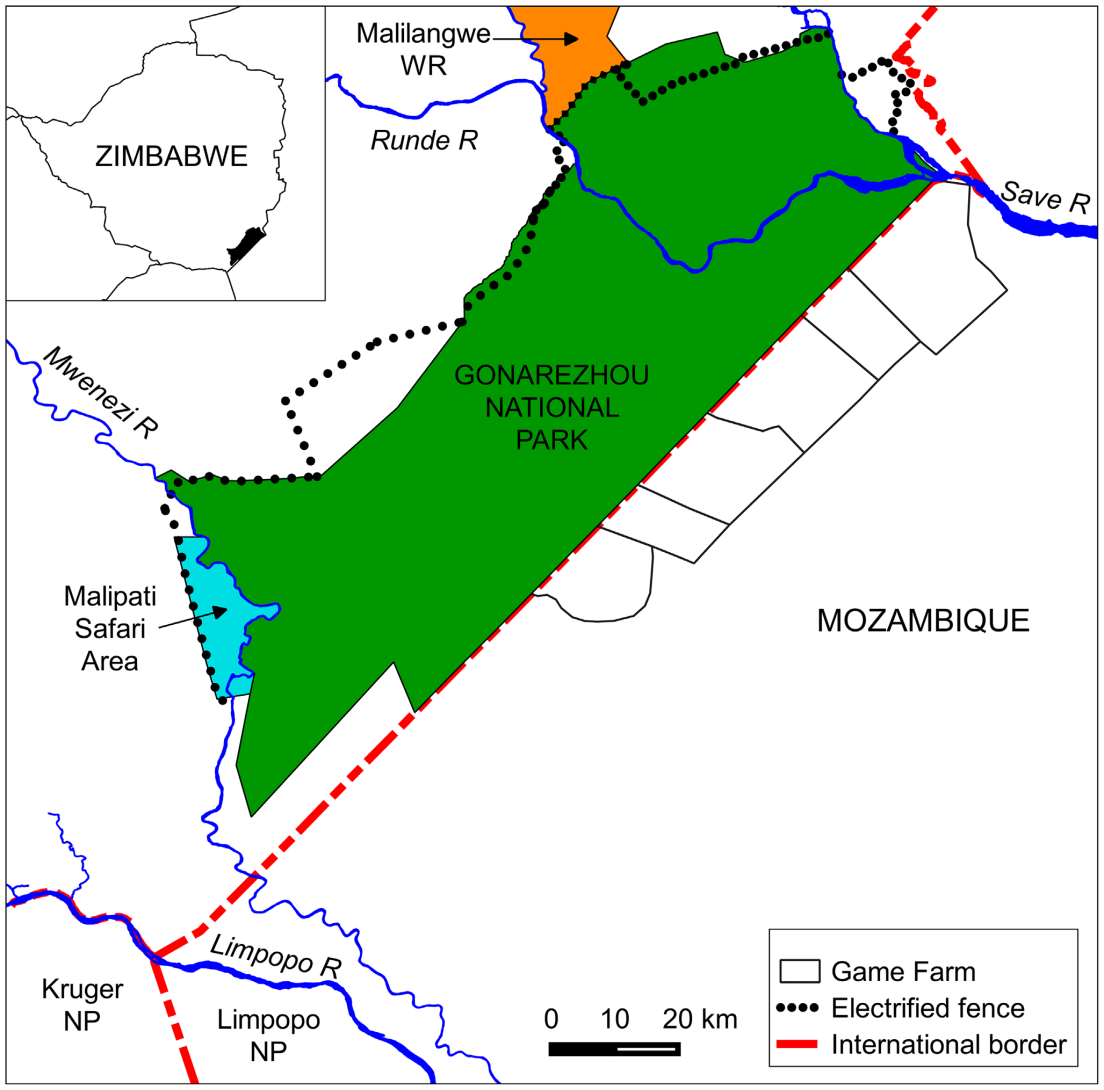
Gonarezhou National Park is in south‐eastern Zimbabwe, adjacent to the international border with Mozambique (insert). South Africa's Kruger National Park (KNP) and Mozambique's Limpopo National Park (LNP) lie to the south.

**FIGURE 2:**
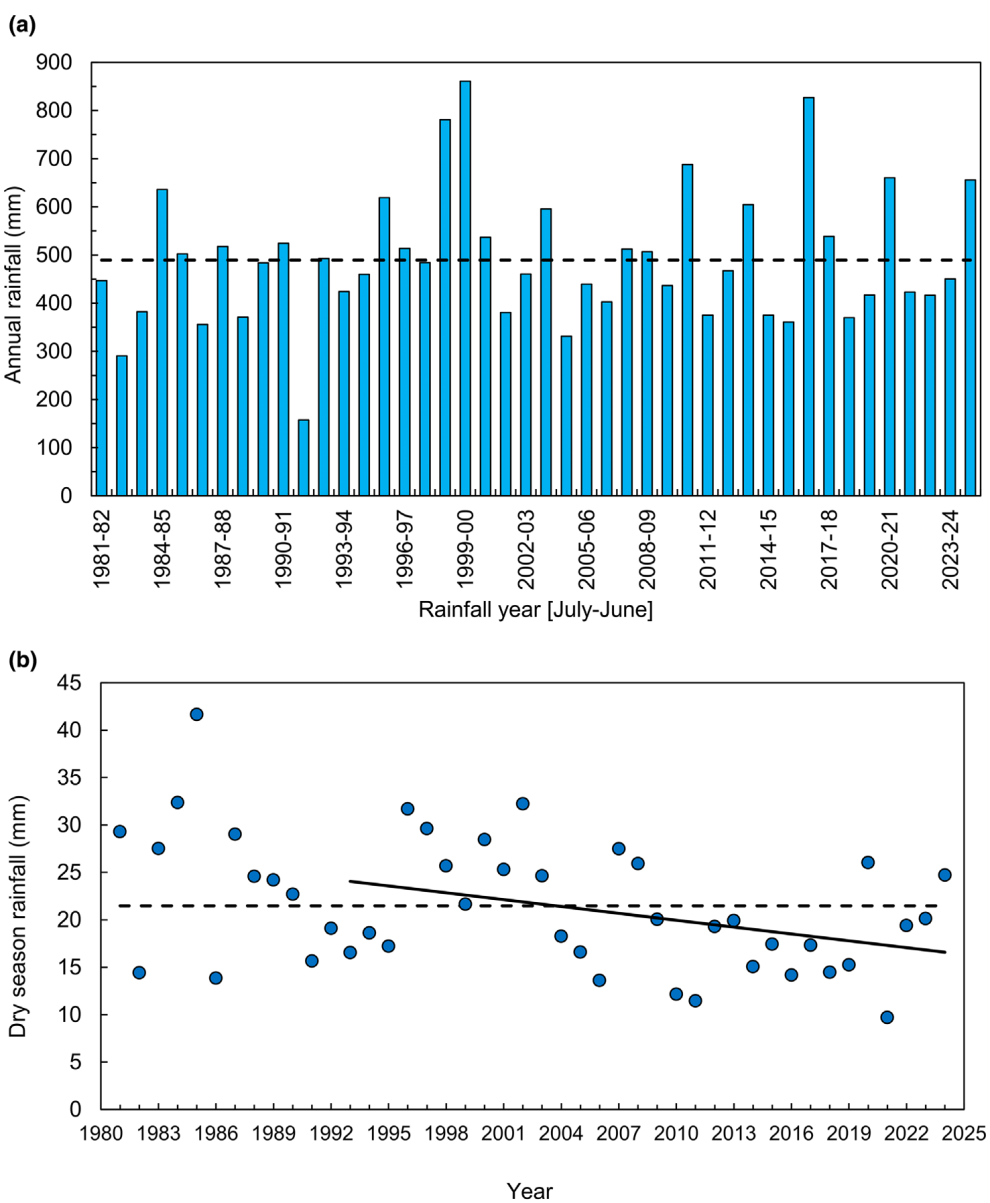
(a) Temporal trend in annual rainfall (July–June) over Gonarezhou NP from 1981 to 2025. The dashed line indicates the mean annual rainfall. (b) Temporal trend in dry season rainfall (June to September inclusive). The dashed line indicates the mean dry season rainfall. The solid line indicates the significant linear trend in dry season rainfall during 1993 to 2024. *Data source:* Climate Hazards Group Infrared Precipitation with Stations (CHIRPS) daily rainfall data downloaded from https://climateserv.servirglobal.net/.

Gonarezhou NP lies < 600 m above sea level and covers ~5000 km^2^ in the south‐east lowveld of Zimbabwe, along the border with Mozambique (Figure 1). The park is within the Great Limpopo Transfrontier Park and the Great Limpopo Transfrontier Conservation Area. South‐eastern Zimbabwe experiences a hot wet season during November–March, a cool dry season during April–July, and a hot dry season during August–October. Mean maximum temperatures exceed 30°C during all months except June and July. The area is noted for low, but very variable, annual rainfall. Based on Climate Hazards Group InfraRed Precipitation with Station (CHIRPS) records (Funk et al. 2015), annual rainfall over the entire park averages 489 mm (July–June rainfall year, *n* = 44 years; coefficient of variation 28%). The drought during 1992 followed a year when annual rainfall was 157 mm (32% of the mean) (Figure 2a). There was no trend in annual rainfall during this study (for rainfall years 1992/1993 to 2024/2025, *F*_1,31_ = 0.22, *p* = 0.6). Nevertheless, annual rainfall, after being close to or above the long‐term mean during 7 of 9 rainfall years immediately following the drought, was below average during 15 of the next 23 years, 2001/2002 to 2024/2025. The medium term variation in annual rainfall may reflect the Dyer‐Tyson cycle, an 18–20 year cycle (~10 years of above‐average rainfall, followed by ~10 years of below‐average rainfall), which has been a feature of rainfall in southern Africa for at least 600 years (Dyer and Tyson 1977; Tyson et al. 2002). Dry season precipitation in the form of drizzle (*guti*) is common in Gonarezhou and averaged 21.5 mm (1981–2025, coefficient of variation 32%) during June–September, but with a significant decline during 1993 to 2024 (*F*_1,30_ = 4.9, *p* = 0.035). Dry season rainfall was above the long‐term average for eight successive years during 1996–2003 inclusive, but below average for 17 of the 21 years during 2004–2024 inclusive (Figure 2b).

**FIGURE 3:**
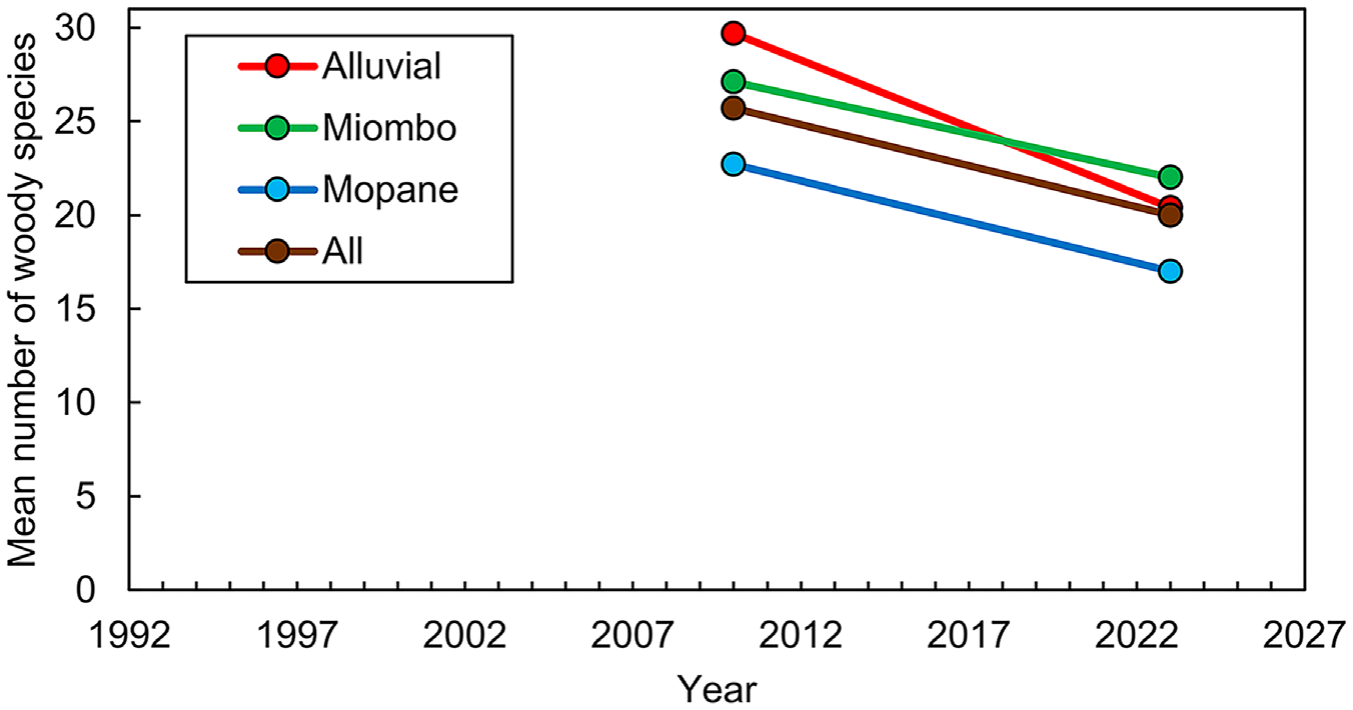
Change in species richness of woody plants per plot between 2010 and 2023 in Gonarezhou NP (All) and within three ecoregions (alluvial, miombo, mopane) of the park. All changes were statistically significant. Data from Mandinyenya et al. (2025).

The Save, Runde and Mwenezi rivers dominate Gonarezhou, although all rise well outside the park. The Runde and Save are perennial, and dry season flow in the Mwenezi is controlled by water release through Manyuchi Dam, ~130 km upstream. Other rivers flow only seasonally. Approximately 40% of Gonarezhou is within 10 km of the Save, Runde or Mwenezi rivers and ~56% is within 15 km. The major vegetation types are *Colophospermum mopane* bushland or woodland on heavier soils; *Guibourtia conjugata* dominated woodland on sands; mixed woodland on granophyre; and riverine woodland (Farrell 1968; Cunliffe et al. 2012). The vegetation has been significantly impacted by elephants, drought and fire (Cunliffe et al. 2012; O'Connor, Ferguson, et al. 2024). Changes in the cover of woody plants have been determined retrospectively from air photographs and satellite images (O'Connor et al. 2026), but there are few quantitative descriptions of temporal changes in vegetation structure and species composition. Mandinyenya et al. (2025) revisited 284 of Cunliffe et al.'s (2012) plots and recorded changes in species richness. Across the entire park, the mean species richness of woody plants declined from 26 to 20 per plot between 2010 and 2023 (Figure 3). The decline was even greater in alluvial areas, where the mean number of woody species declined from 30 to 20 per plot. Although changes in woody species composition were weakly related to fire frequency, elephant use—although not included as a possible explanatory factor in the analysis—was believed to be primarily responsible (Mandinyenya et al. 2025).

**FIGURE 4:**
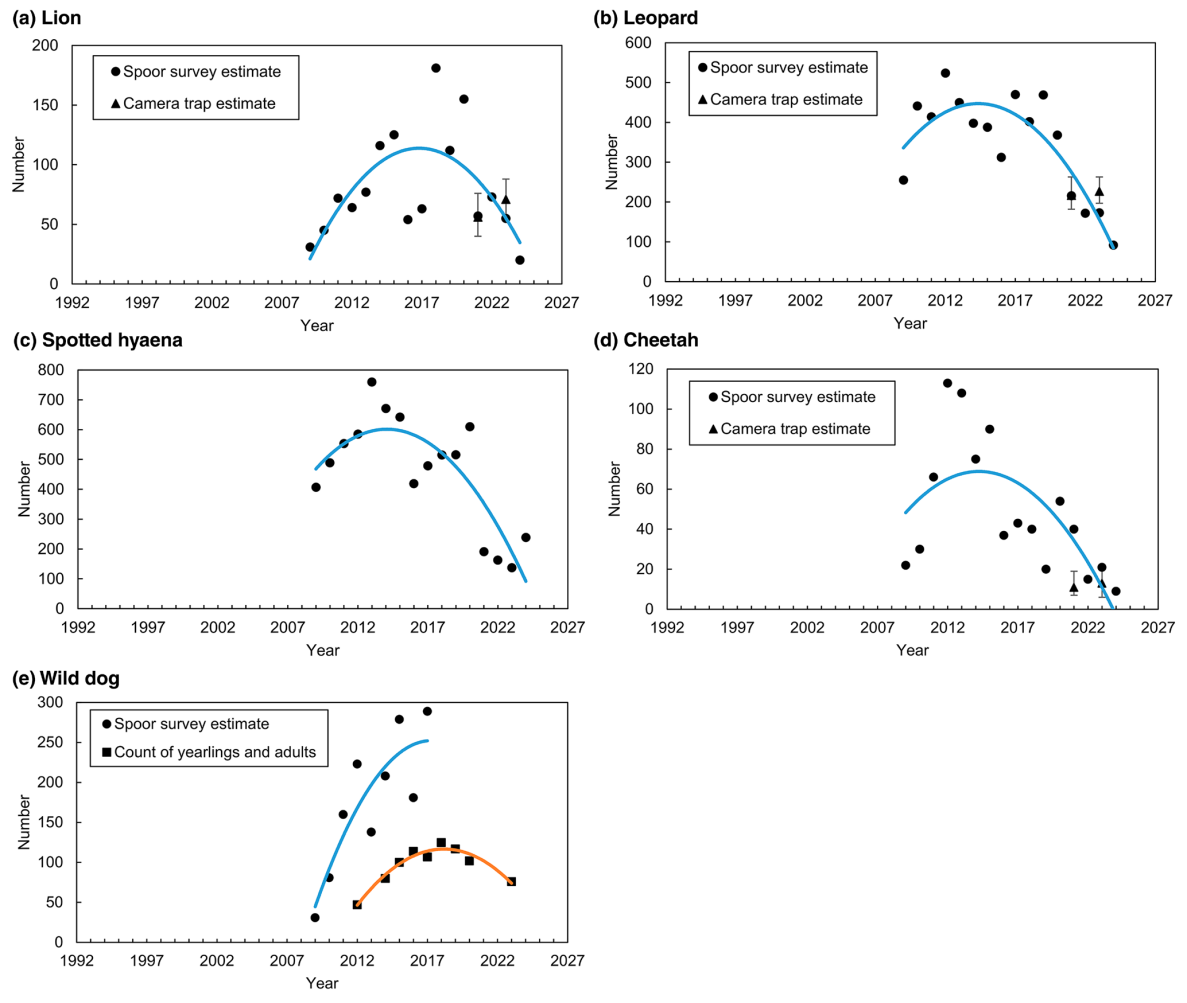
Temporal trends in the estimated numbers of large predators in Gonarezhou during 2009–2024: (a) lion; (b) leopard; (c) spotted hyaena; (d) cheetah; and (e) wild dog. Solid circles are spoor survey estimates; solid triangles are camera trap survey estimates; and solid squares are counts of identified individual yearlings and adults. Vertical bars indicate maximum and minimum estimates. Temporal trends are fitted as 2‐order polynomials and are for illustrative purposes only: blue lines for spoor survey estimates and orange line for counts of individuals. Camera trap survey data are from Loveridge et al. (2024) and all other data are from the African Wildlife Conservation Fund (Groom and Watermeyer 2017; Watermeyer and Groom 2022; African Wildlife Conservation Fund 2023; Shito and Watermeyer 2024).

As well as elephant, Gonarezhou supports populations of black rhinoceros *Diceros bicornis* , white rhinoceros *Ceratotherium simum simum* , hippopotamus *Hippopotamus amphibius* , buffalo, zebra, ostrich, a variety of antelope species, and large predators. There are no data on predator populations in Gonarezhou for 1992–2008. Predator spoor counts during 2009–2024 (Groom and Watermeyer 2017; Watermeyer and Groom 2022; African Wildlife Conservation Fund 2023; Shito and Watermeyer 2024) indicate broad population trends (Funston et al. 2010), with wild dog estimates supplemented by counts of identified individual yearlings and adults. Only recently have camera surveys provided robust estimates of predator numbers (Loveridge et al. 2022, 2024). The estimated numbers of lion, leopard, spotted hyaena, wild dog and cheetah in Gonarezhou all increased after 2009 and then peaked, before declining to low levels by 2023–2024 (Figure 4). Trend lines indicating the temporal changes in predator numbers suggest population number peaked during approximately 2014 for leopard, spotted hyaena and cheetah and 2017/2018 for lion and wild dog.

In response to food shortages during the 1992 drought, some animals were captured and removed from the park; and some elephants were culled (Dobb 1993; Tayler 1992). Many other animals died, but there are no reliable estimates of the numbers that died. During the drought, water was pumped to what would otherwise be seasonal pans, but pumping ended soon after the drought. During 1993, as an alternative to culling, 670 elephants in complete female herds were captured and translocated elsewhere (Coetsee 1996).

During 1998–2000, ~600 buffalo from northern Zimbabwe were translocated to Gonarezhou (Smitz et al. 2014; Cumming 2016). Later, large herbivores from Sango Ranch (in Save Valley Conservancy) or Malilangwe Wildlife Reserve (WR) were moved to Gonarezhou, including 100 buffalo during 2012 and 34 giraffe, 96 eland, 97 zebra, 100 buffalo, 376 impala and 390 wildebeest during 2013 (Dunham et al. 2013). Anthrax killed many greater kudu in Malilangwe WR during 2004 (Clegg et al. 2007) and at least 128 animals, including 75 buffalo, 38 kudu and 4 elephant, in southern Gonarezhou during 2024 (Dube 2024). The Malilangwe outbreak started in the north of the property during mid August 2004 and spread southwards, reaching the southern boundary (with Gonarezhou) in mid September (Clegg et al. 2007). In Malilangwe, kudu was the species most affected (569 carcasses found, representing ~100% of the previous year's population estimate), but others included nyala (34 carcasses, 68%), waterbuck (36 carcasses, 44%) and buffalo (92 carcasses, 9%). For giraffe, eland, warthog and zebra, the number of carcasses represented 3%–9% of the population estimate. Impala was the least affected large herbivore species (21 carcasses, 0.5%). No elephant or wildebeest carcasses were found. Overall, browsers accounted for 78% of herbivore mortalities, while grazers and mixed feeders accounted for only 22%. The number of carcasses found in Malilangwe declined noticeably after the first rains (24 mm on 10 October at Chipinda Pools station in northern Gonarezhou), with the last deaths during early November. The Malilangwe/Gonarezhou boundary fence was not completely effective, but there are no available records to indicate whether the outbreak spread to Gonarezhou.

Since 2011–2012, electric fences have limited the movement of large mammals to the north and north‐west of Gonarezhou and, since 2016, the fence line has been extended southwards along the western boundary. The movement of large mammals between Gonarezhou and Malilangwe has been very limited since the latter's boundary fence was completed during 2011 (B. Clegg, pers. comm., 3 January 2023). Within Mozambique, adjacent to the unfenced international border, are *fazendas de bravio* (game farms—privately‐managed areas intended for both wildlife conservation and hunting) totalling ~1400 km^2^. Along the south‐western border of Gonarezhou, across the Mwenezi River, is Malipati Safari Area, where safari hunting occurred until 2017. To the north‐east of Gonarezhou, across the Save River, is Mahenye communal area, where a dedicated wildlife area was fenced during 2017. Since 2012, some elephants have moved into Malipati and Mahenye and some across the international border into Mozambique (Dunham 2025a).

Coincident with the occupation of Zimbabwean commercial farms during 2000, people from the Chitsa community illegally occupied an area of ~90 km^2^ in northern Gonarezhou and grazed cattle in and around this area (Mombeshora and Le Bel 2009; Gandiwa, Matsvayi, et al. 2011). But by 2013, this area had been fenced from the remainder of the park. Hunting of large herbivores by park staff for rations used to occur in Gonarezhou, but ceased after the Frankfurt Zoological Society (FZS) started to assist park management during 2007. Since 2017, the park has been managed by the Gonarezhou Conservation Trust (GCT), a joint venture between Zimbabwe's Parks and Wildlife Management Authority and FZS. Some illegal hunting of wildlife occurs in the park.

### 2.2 Aerial Surveys

Sample aerial surveys of the Gonarezhou elephant population have been undertaken regularly during the dry season by the Parks and Wildlife Management Authority (PWMA), often in partnership with the World Wide Fund for Nature (WWF), FZS, or GCT. The methods were those recommended for surveys of large African herbivores (Jolly 1969; Norton‐Griffiths 1978; Craig 2012). Surveys with stratification (six strata since 1995) and systematically arranged transects were conducted in 14 years during 1993–2025 (Table 1). Importantly, the survey techniques have been consistent since the start of the survey programme during 1980. The reports of the post‐drought surveys are publicly available (Bowler 1995; Davies 1996; Davies et al. 1996; Mackie 1999; Dunham 2002, 2022, 2025b; Dunham et al. 2007, 2010, 2013, 2022; Dunham and van der Westhuizen 2015, 2016, 2018).

The Gonarezhou aerial surveys were designed to produce reasonably accurate and precise estimates of elephant numbers. In their experiments, Schlossberg et al. (2016) found that observers detected 87% of individual elephants during such surveys. Other large herbivore species were also recorded during the Gonarezhou surveys, even though some are not easily seen from the air and their numbers are undoubtedly underestimated. Jachmann (2002) found that animals that moved in response to an aircraft were more likely to be seen than static ones; dark‐coloured animals were easier to spot than light‐coloured ones against a light background; large herds were easier to detect than small ones; and large animals were more easily seen than small ones. The current study does not assume that the population estimates derived from the aerial surveys are accurate. Instead, it assumes that the population estimates provide indices of abundance, with measures of precision, that can be used to determine temporal trends in population number.

The hippopotamus was not included in this study, because aerial sample surveys are generally not suitable for this species, which is better monitored using dedicated aerial total counts along perennial rivers and waterbodies (e.g., Zisadza et al. 2010). The rhinoceroses were also omitted from the study, because aerial surveys are not a good technique to monitor rhinoceros numbers (Goddard 1969). The rhinoceroses were reintroduced to Gonarezhou only recently (Capon et al. 2025) and as the individuals are relatively few in number and individually recognisable, they are more efficiently monitored by ground patrols.

### 2.3 Data Analysis

Data on large herbivores (species listed in Table 1) were extracted from the survey reports. The population estimates and variances given in the survey reports had been calculated using Jolly's (1969) method 2. No corrections were applied to compensate for any undercounting or missed animals. For consistency, for surveys during 1993–1998, 95% confidence limits were recalculated for all mean estimates of population number as follows: Population estimate ± (*t*_*v*_. √Total Variance); where *t* = Student's *t*, and *v* = degrees of freedom estimated by Satterthwaite's rule (Snedecor and Cochran 1980). If, for any species and survey, the calculated lower confidence limit was less than the number of individuals seen in the search strips, this number was substituted for the lower confidence limit.

Separate estimates of the numbers of elephants in female herds and in bull groups were available. Elephants in female herds included adult females, immature elephants of both sexes and probably a few adult bulls. Bull groups contained subadult and adult males. The ostrich was included in surveys because, being large and herbivorous, it was ecologically similar to the large mammalian herbivores. The surveys were not intended to provide population estimates for small antelopes, but nevertheless estimates are available for common duiker and steenbok (Table 1). The assumption that data from the elephant surveys provide accurate estimates of real trends in the populations of other large herbivores depends on the visibility bias of these species remaining approximately constant during the survey programme. Elephants have had a major impact on the vegetation in Gonarezhou, for example, reducing the density and average height of trees in *Colophospermum mopane* woodland, and transforming species‐rich, closed‐canopy riverine woodland into depauperate open woodland (O'Connor, Pallett, et al. 2024; O'Connor et al. 2026). As is shown below, the survey estimates for the smaller herbivores declined during the latter part of the survey programme. But if the survey estimates for the smaller herbivores changed simply because individuals of these species became more visible to survey observers as a result of elephant opening up the woody vegetation, the opposite trend (an increase in estimates) would be expected.

The analyses of population trends were adjusted for some species if surveys returned zero estimates, or if relatively large numbers of animals were released in the park (details in Table 1). The numbers of buffalo, giraffe, impala and zebra released during 2012–2013 represented just 3%–7% of the 2013 survey estimates for these species (Dunham et al. 2013), and the impact of these releases on the population trends of these species may have been slight, or even negligible. In any case, the percentage confidence intervals of the survey estimates for these species were greater than 7%, which would have limited the ability of the surveys to detect any impacts.

All time series of survey estimates were graphed to see if there appeared to be trends in the estimates. During any period when the rate of change in estimates appeared to be approximately constant, the trend in animal numbers was determined using an exponential model to estimate the rate of population change. The exponential rate of population change per annum (*r* hat) was calculated using a method based on a weighted regression of natural logarithms of the survey estimates against time, with the variance of *r* based on the sampling variances of the survey estimates. The percentage rate of population change per annum was: 100 · (e^*r*^ − 1). This technique is the one suggested by Gasaway et al. (1986) for determining the rate of population change from survey estimates when the exponential model of population change is appropriate. The variance of *r* was small when the sampling variances were small, reflecting the greater confidence that could be placed in a value of *r* calculated from precise estimates. The time interval between successive surveys varied from 1 to 6 years, reflecting the logistic and financial constraints that are common to African wildlife survey programmes. The impact, if any, of the varying sample interval on the determined trends was reduced by calculating each rate of population change only for a period when a plot of the survey estimates suggested that the rate was constant. Some species exhibited a period of increasing survey estimates, followed by a period of declining estimates. The overall trend for these species was described with two separate trend lines, one for the period of increase and a second for the decline. A population was considered to have increased significantly if both the lower and upper 95% confidence limits of *r* were positive, or to have declined if both limits were negative.

When a plot of survey estimates suggested that the rate of population change (but not the estimates) decreased with time, a Ricker logistic curve (Sibly et al. 2005; Koetke et al. 2020) was fitted to the estimates. The equation for this curve describes the population growth using three parameters: *r*_m_ is the population growth rate when the population number (*N*) is at a low value, and *K* is the population's carrying capacity: *N*_*t*+1_ = *N*_*t*_ · exp(*r*_m_(1 − (*N*_*t*_/*K*))). The MS Excel add‐in, Solver *Evolutionary*, was used to determine the starting values for *N*_93_ (population number during 1993), *r*_m_ and *K* that resulted in the modelled number of a given species providing the closest fit to all the survey estimates for years when the Gonarezhou population of that species was actually censused. The starting values for the three unknowns varied between species. *N*_93_ was initially set to be the same as the 1993 (or 1995) survey estimate, with its constraints set the same as the confidence limits for this estimate. The growth rate, *r*_m_, was initially set to be 0.1, 0.15, or 0.2 (with a smaller value for large species) and with constraints 0 and 0.5. The carrying capacity, *K*, was initially set to be an integer approximately equal to the mean of the post‐2013 survey estimates for that species, with its constraints set to be similar to the smallest and largest confidence limits of these estimates.

Running Solver was equivalent to running numerous models with different starting values for the three unknowns. Each model was assessed by comparing the fit of the predicted animal numbers with the survey estimates. Martin's estimator (Martin 1992; Dunham 2008) was used to compare the fit of the different models. Each survey estimate was compared with the predicted population number for that year by relating the difference between the two to the variance of the survey estimate. There were 14 aerial surveys of the Gonarezhou animal populations during 1993–2025 and usually each provided an estimate of the number of a given species. For each combination of the three unknowns, the product of the 14 comparisons of predicted population number with survey estimate gave a single ‘index value’, which was minimised using Solver. For species with less than 14 survey estimates, the number of comparisons was equal to the number of estimates.

The Solver *Evolutionary* can find a sound solution, but not necessarily one which is optimal (Frontline Systems 2026). To increase the likelihood that Solver found a solution close to optimal, the default options were changed, with the convergence reduced (to 0.000001; for giraffe to 0.0000001), the mutation rate increased (to 0.075) and the maximum time without improvement increased (to 600 s; for giraffe to 1000 s). Solver found an identical solution even if the initial values for the three unknowns were changed, either to their minimum or maximum constraints.

Values for *r*_m_, *N*_93_ and *K* were determined simply to calculate logistic curves that best fit the survey estimates for those species that did not decline in abundance after 2013/2014. The curves were for illustrative purposes only and no biological significance was attached to these values. Hence, variation in the time interval between successive surveys, even if it affected the calculated values, was not a concern.

## 3 Results

**FIGURE 5:**
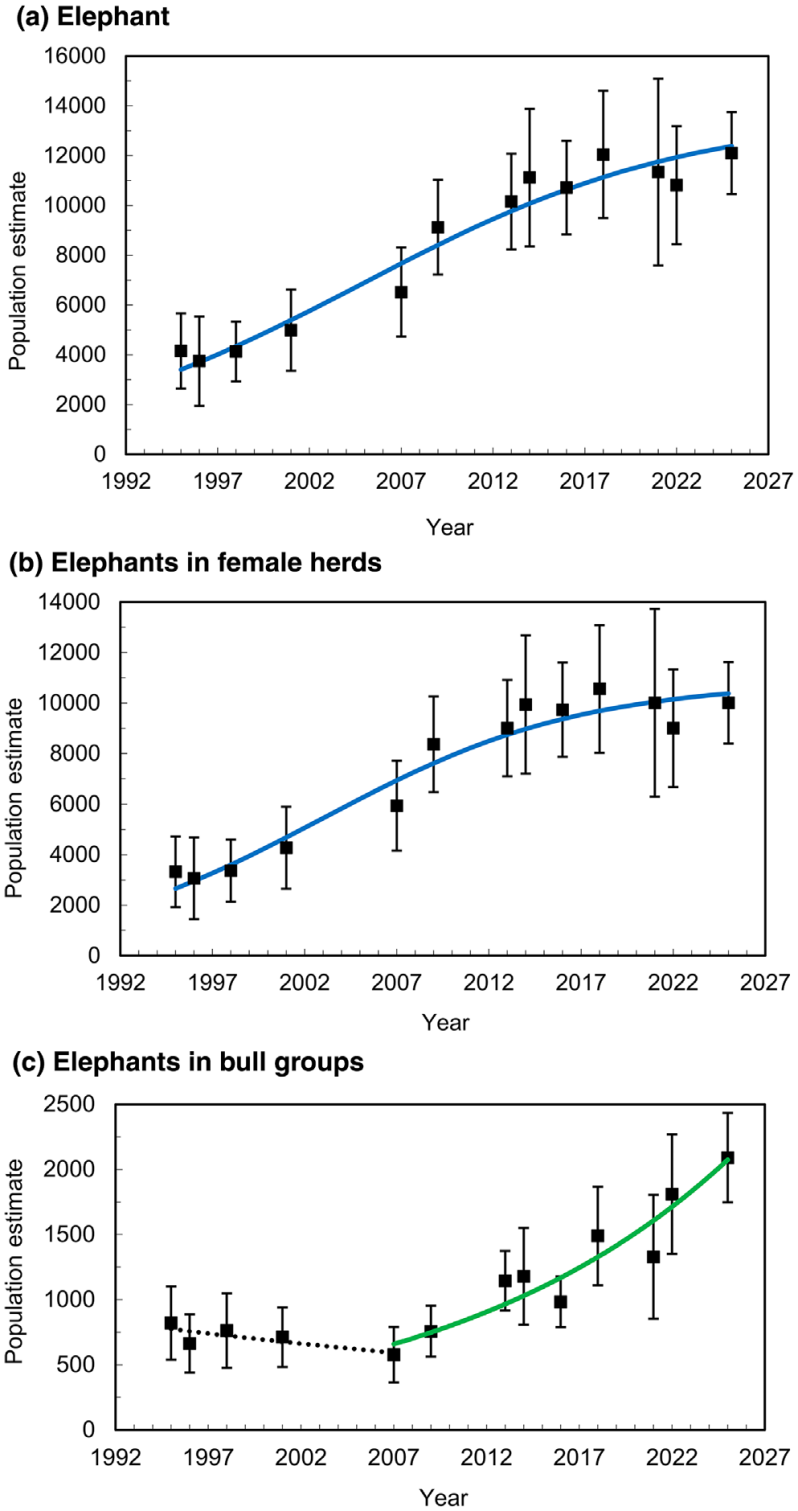
(a) Temporal trend in the estimated number of elephants in Gonarezhou. Points are survey estimates with 95% confidence intervals. Blue line is logistic curve with parameters *N*_93_ = 2860, *r*_*m*_ = 0.112 and *K* = 13,661. (b) Temporal trend in the estimated number of elephants in female herds. Blue line is logistic curve with parameters *N*_93_ = 2130, *r*_*m*_ = 0.142 and *K* = 10,827. (c) Temporal trend in the estimated number of elephants in bull groups. Dotted line indicates non‐significant trend and green line indicates significant exponential trend (Table 3).

**FIGURE 6:**
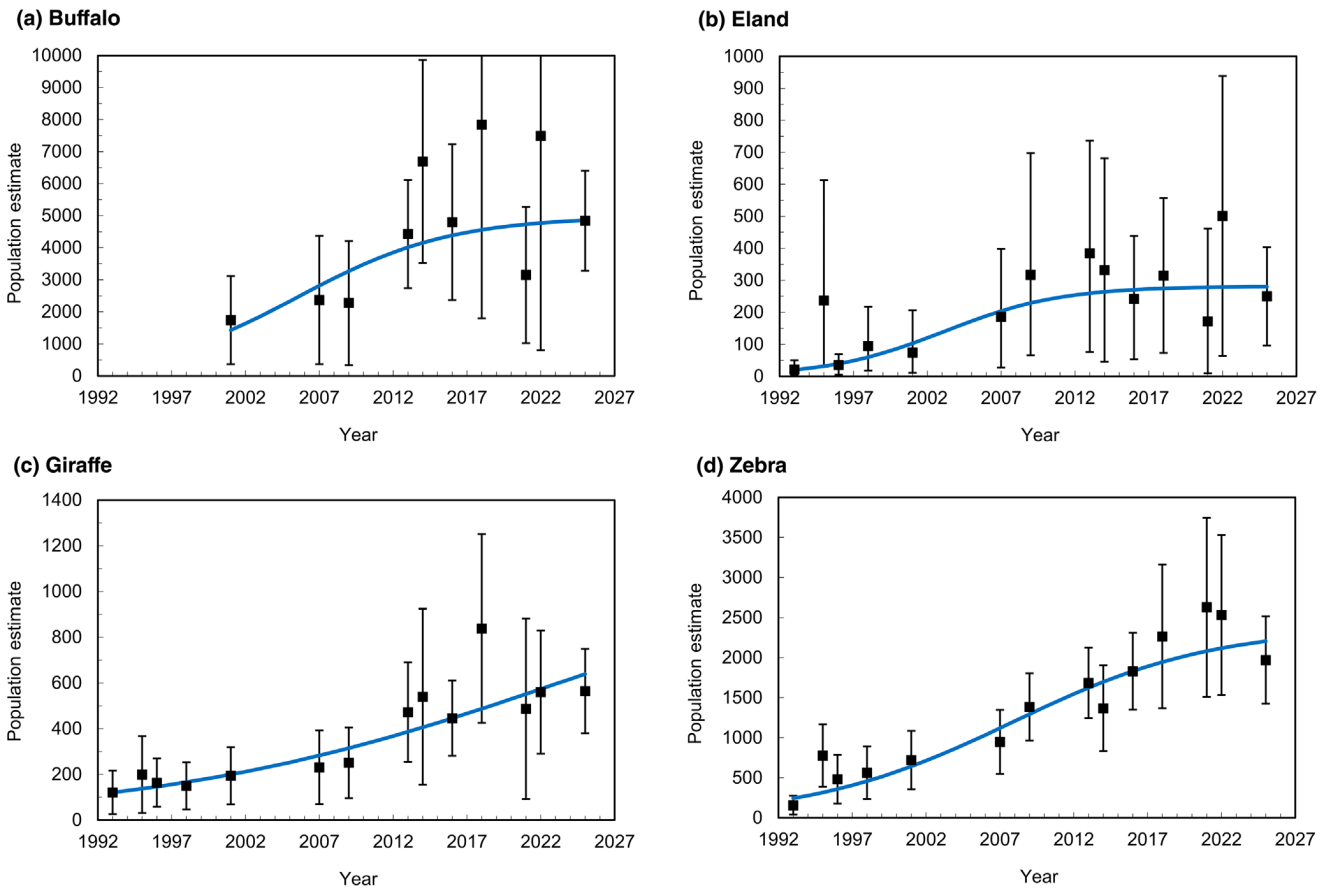
Temporal trends in the estimated numbers of four species of large herbivore which did not decline in abundance in Gonarezhou after 2013: (a) buffalo; (b) eland; (c) giraffe; and (d) zebra. Points are survey estimates with 95% confidence intervals. Blue lines are logistic curves. Parameters of curves are: *N*_93_ = 388, *r*_*m*_ = 0.195 and *K* = 4968 for buffalo; *N*_93_ = 20, *r*_*m*_ = 0.253 and *K* = 281 for eland; *N*_93_ = 121, *r*_*m*_ = 0.072 and *K* = 1222 for giraffe; and *N*_93_ = 244, *r*_*m*_ = 0.147 and *K* = 2384 for zebra.

**FIGURE 7:**
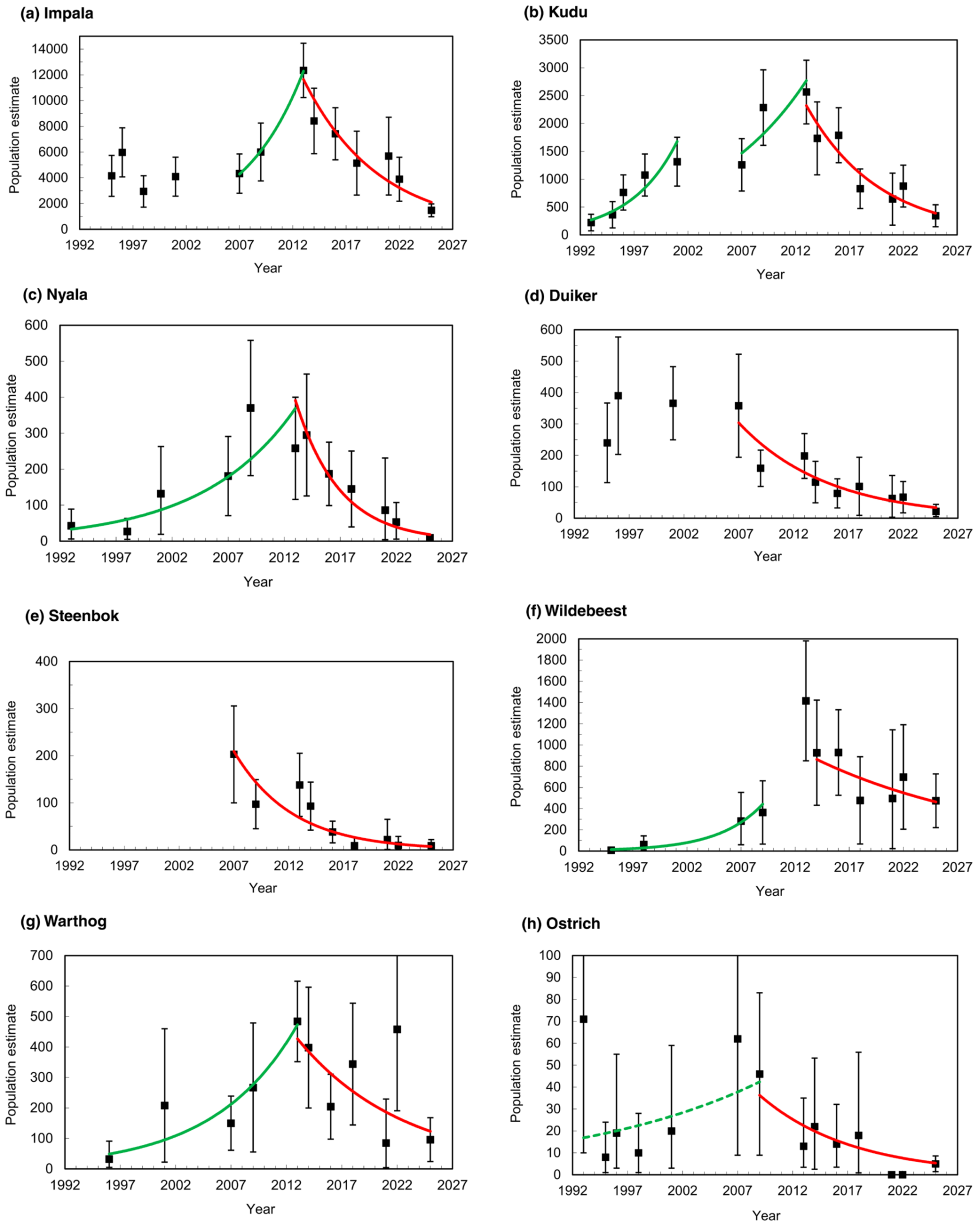
Temporal trends in the estimated numbers of eight species of large herbivore which declined in abundance in Gonarezhou after 2013: (a) impala; (b) kudu; (c) nyala; (d) duiker; (e) steenbok: (f) wildebeest; (g) warthog and (h) ostrich. Points are survey estimates with 95% confidence intervals. Bold lines are exponential curves representing significant rates of increase (green lines) or decrease (red lines) (Table 2). Dashed green line is exponential curve, with rate of increase significant at *p* = 0.1 (Table 2). Duiker and steenbok declined in abundance after 2007, and ostrich after 2007 or 2009.

During ~20 years after the 1992 drought, the estimated numbers of elephant (and elephants in female herds), buffalo, eland, giraffe, kudu, nyala, warthog, waterbuck, wildebeest, zebra and probably ostrich in Gonarezhou increased (Figures 5, 6, 7). The estimate for impala was approximately constant initially, but increased after 2007 (Figure 7a). All these increases were statistically significant (Tables 2 and 3).

**TABLE 2:** Trends in the estimated numbers of large herbivores in Gonarezhou NP after the 1992 drought.

Species	Years	Number of surveys	*r*	% change per annum	LCL *r*	UCL *r*	Trend^a^
Common Duiker *Sylvicapra grimmia*	2007–2025	9	−0.113	−10.7	−0.149	−0.077	−
Savannah elephant *Loxodonta africana*	1995–2014	8	0.058	6.0	0.044	0.072	+
Impala *Aepyceros melampus*	1995–2007	5	−0.010	−1.0	−0.046	0.026	ns
	2007–2013	3	0.176	19.2	0.118	0.234	+
	2013–2025	7	−0.155	−14.4	−0.182	−0.128	−
Kudu *Tragelaphus strepsiceros*	1993–2001	5	0.190	20.9	0.117	0.262	+
	2007–2013	3	0.090	9.4	0.026	0.153	+
	2013–2025	7	−0.149	−13.8	−0.185	−0.113	−
Nyala *Tragelaphus angasii*	1993–2013	6	0.108	11.4	0.060	0.155	+
	2013–2025	7	−0.226	−20.2	−0.305	−0.147	−
Ostrich *Struthio camelus*	1993–2009	7	0.064	6.7	−0.012	0.141	ns^a^
	2009–2025	6^c^	−0.142	−13.2	−0.243	−0.040	−
Steenbok *Raphicerus campestris*	2007–2025	9	−0.162	−15.0	−0.213	−0.112	−
Warthog *Phacochoerus africanus*	1996–2013	5	0.133	14.2	0.075	0.190	+
	2013–2025	7	−0.082	−7.9	−0.130	−0.034	−
Waterbuck *Kobus ellipsiprymnus*	1993–2013	8	0.162	17.6	0.114	0.210	+
	2013–2025	7	−0.049	−4.8	−0.112	0.013	ns^b^
Wildebeest *Connochaetes taurinus*	1995–2009	4	0.224	25.1	0.129	0.319	+
	2014–2025	6	−0.062	−6.0	−0.120	−0.004	−

*Note:* For buffalo, eland, elephant, giraffe and zebra, the trend in the entire series of survey estimates is represented by a logistic curve (see Figure 6).

Abbreviations: +, significant increase in estimated population number; −, significant decrease in estimated number; LCL *r*, lower 95% confidence limit of *r*; ns, no statistically significant trend; *r*, exponential rate of population change per annum; UCL *r*, upper 95% confidence limit of *r*.

^a^ There is a significant increase if using 90% confidence limits of *r*, instead of 95% confidence limits.

^b^ There is a significant decrease if using 87% confidence limits of *r*, instead of 95% confidence limits.

^c^ Zero estimates during 2021 and 2022 are excluded from the calculation of the rate of population change *r*.

**TABLE 3:** Trends in the estimated numbers of elephants in female herds and of elephants in bull groups in Gonarezhou NP after the 1992 drought.

Species	Years	Number of surveys	*r*	% change per annum	LCL *r*	UCL *r*	Trend
Elephants in female herds	1995–2014	8	0.064	6.6	0.047	0.080	+
Elephants in bull groups	1995–2007	5	−0.022	−2.1	−0.058	0.015	ns
	2007–2025	9	0.062	6.4	0.049	0.076	+

*Note:* For elephants in female herds, the trend in the entire series of survey estimates is represented by a logistic curve (see Figure 5).

Abbreviations: +, significant increase in estimated population number; LCL *r*, lower 95% confidence limit of *r*; ns, no statistically significant trend; *r*, exponential rate of population change per annum; UCL *r*, upper 95% confidence limit of *r*.

After 2014, the rate of change in the survey estimates for elephant and for elephants in female herds declined noticeably, but was not negative, and so logistic curves were fitted to the survey estimates (Figure 5a,b). A logistic curve was a credible fit to the survey estimates for the number of elephants in female herds, but the survey estimates themselves suggested that there may have been an abrupt decrease in the rate of population increase, with 2014 being a pivotal year (Figure 5b). The estimated number of elephants in female herds varied little after 2018, averaging 9895 during 2018–2025. The trend in the survey estimate for elephants in bull groups was unusual: from 1995 the estimate was constant or declined slightly, but after 2007 it increased exponentially (Figure 5c).

After approximately 2013/2014, the rate of change in the survey estimates decreased for buffalo, eland, giraffe and zebra, but was not negative, suggesting that a logistic curve was an appropriate model for the entire series of survey estimates for these species. For buffalo and eland, the survey estimates after 2013/2014 were variable and imprecise and the fitted logistic curves for these species imply that the survey estimate was approximately constant during the latter part of the study (Figure 6a,b). In contrast, the fitted logistic curves for giraffe and zebra suggest that the populations were still increasing during the latter part of the study, although at a slower rate than previously (Figure 6c,d).

**FIGURE 8:**
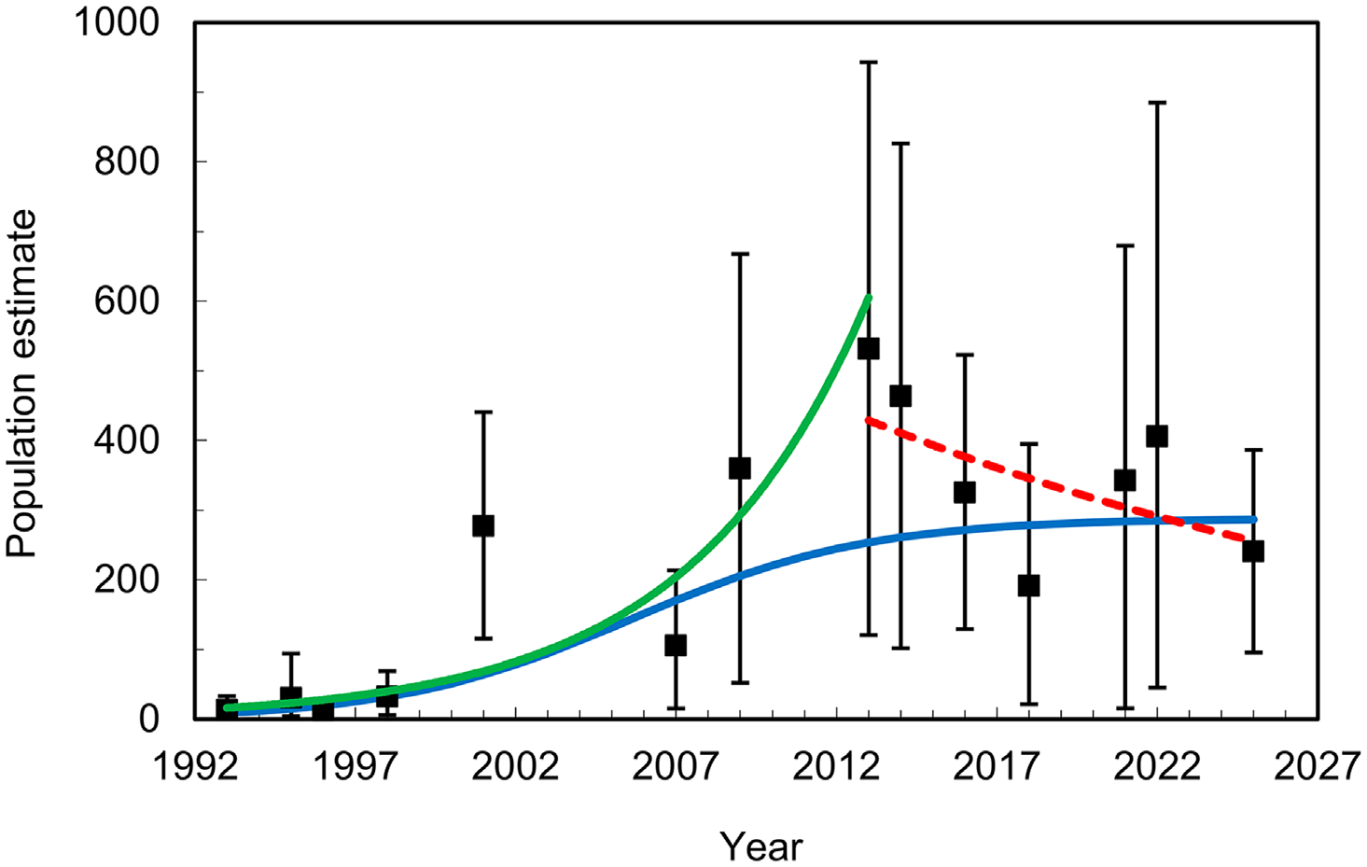
Temporal trends in the estimated number of waterbuck in Gonarezhou. Points are survey estimates with 95% confidence intervals. It is unclear whether the overall trend is best represented by a: Logistic curve (blue line) with the parameters *N*_93_ = 9, *r*_*m*_ = 0.272 and *K* = 288; or two exponential curves, one showing a period of increase (green line) and the second a period of decrease (dashed red line), although the apparent decline was not statistically significant (Table 2).

For impala, kudu, nyala, warthog and wildebeest, the survey estimates declined significantly after 2013 and for two smaller antelopes, duiker and steenbok, the estimates declined after 2007 (Figure 7, Table 2) (although there are no pre‐2007 estimates for steenbok). For ostrich, the survey estimates declined after 2009 (or 2007) (Figure 7h). For waterbuck, an apparent decline in the estimates after 2013 was not statistically significant (*p* = 0.13) (Table 2). Hence, it is unclear for waterbuck if the entire series of survey estimates is best modelled by a logistic curve, or by two exponential lines, one modelling the pre‐2013 increase and another modelling a post‐2013 decline (Figure 8).

There was no association between the degree of dependence on drinking water of a species (Table 4) and whether its population declined after 2013. The species that declined included water dependent species (e.g., impala, warthog, wildebeest) and water independent species (duiker and steenbok). The species that did not decline also included water dependent species (buffalo and zebra) and water independent species (eland and giraffe). Also, there is no association between the feeding style of a species (Table 4) and whether its population declined after 2013. The species that declined included grazers (warthog and wildebeest), browsers (duiker, kudu and steenbok) and mixed feeders (impala and nyala). The species that did not decline also included grazers (buffalo and zebra), browsers (giraffe) and mixed feeders (elephant). However, for those species which did decline, the population estimates for mammalian browsing and mixed feeding species (kudu, nyala, impala, duiker and steenbok) declined at 11%–20% per annum, faster than the estimates for mammalian grazers (warthog and wildebeest), which decreased at 6%–8% annually.

**TABLE 4:** Diet, size and water dependence of large herbivore species recorded during aerial surveys of the elephant population in Gonarezhou National Park.

Species	Feeding style	Unit body mass (kg)^a^	Water dependence^b^
Buffalo *Syncerus caffer*	Grazer^c^	450	High
Common Duiker *Sylvicapra grimmia*	Browser^c^	10	None
Eland *Tragelaphus oryx*	Mixed, but predominantly Browser^c^	340	None
Savannah elephant *Loxodonta africana*	Mixed^d^	1725	High
Giraffe *Giraffa camelopardalis*	Browser^c^	750	Low
Impala *Aepyceros melampus*	Mixed^c^	40	High
Kudu *Tragelaphus strepsiceros*	Browser^c^	136	Low
Nyala *Tragelaphus angasii*	Mixed^c^, ^e^	73	Low
Ostrich *Struthio camelus*	Mixed^f^	69^g^	Low^h^
Steenbok *Raphicerus campestris*	Browser^c^	10	None
Warthog *Phacochoerus africanus*	Grazer^i^	45	High
Waterbuck *Kobus ellipsiprymnus*	Grazer^c^	160	High
Wildebeest *Connochaetes taurinus*	Grazer^c^	123	High
Zebra *Equus quagga*	Grazer^j^	200	High

*Note:* Mixed feeders eat both browse (woody and non‐woody dicotyledonous plants) and grass, with their diets often varying seasonally and grass eaten mainly during the rainy season.

^a^ Coe et al. (1976).

^b^ Hempson et al. (2015).

^c^ Sponheimer et al. (2003).

^d^ Guy (1976).

^e^ Lobão Tello and Van Gelder (1975).

^f^ Milton et al. (1994).

^g^ Maclean (1985).

^h^ Williams et al. (1993).

^i^ Cumming (1975).

^j^ Jarman (1971).

There was an association between the body size of a species (Table 4) and whether its population declined after 2013. The populations that did not decline were the larger species, namely zebra, eland, buffalo, giraffe and elephant. The populations that declined were the smaller of the studied species, namely wildebeest, kudu, nyala, warthog, impala, duiker, steenbok and ostrich. The population trend of waterbuck is uncertain: it has a body mass (160 kg) intermediate between those of kudu (136 kg), which declined in number, and zebra (200 kg), which did not.

## 4 Discussion

Elephants dominate the ecology of Gonarezhou and first the trend in elephant number since the 1992 drought is considered. Then how management and disease may have affected the trends of other large herbivore species during the two decades after the drought is reviewed; followed by consideration of the factors that may have caused the populations of some large herbivore species, but not others, to decline after 2013.

### 4.1 Elephant

The effect of the 1992 drought on elephant numbers in Gonarezhou during 1995–2022 has already been described (Dunham 2025a). The 2025 survey estimates confirmed that: (a) the number of elephants in female herds had approximately levelled off, and had not declined recently as perhaps suggested by the 2022 estimate; and (b) the number of elephants in bull groups continued to increase exponentially. The high mortality of immature elephants, particularly males, during the 1992 drought caused the number of bulls recruited to the bull groups from the female herds to remain low for ~16 years after the drought (~16 years being the approximate age when males leave the female herds and move to the bull groups). But at the end of that period, ~16 years after the drought, the number of elephants in bull groups increased exponentially at a mean rate of 6.4% per annum reflecting a similarly high rate of increase (6.6% per annum) in the number of elephants in female herds some 16 years earlier.

The levelling‐off of the number of elephants in female herds inside Gonarezhou coincided with the movement of some female herds out of the park and into adjacent areas after 2012. But these movements accounted only partly for the levelling‐off, which occurred without any noticeable increase in adult mortality and thus likely reflects a decline in the birth rate, or juvenile survival rate, or both (Dunham 2025a).

### 4.2 Population Trends of Other Species 1993–2013

The Gonarezhou populations of buffalo, eland, elephant, giraffe, impala, kudu, nyala, warthog, waterbuck, wildebeest, zebra and probably ostrich increased in number during the two decades after the 1992 drought. There are no reliable estimates of large herbivore mortality during that drought, but contemporary accounts suggest that it was high (Tayler 1992). Thus, it is not surprising that the estimates of the numbers of large herbivores in Gonarezhou during 1993 and 1995, 1–3 years after the drought, were small and that generally there were substantial increases in the estimates during the two decades following it. The 1992 drought was followed by a decade when both annual rainfall and dry season rainfall were above average or approximately average, and so it is likely that, during this period, food was abundant for the relatively small numbers of drought survivors.

The estimated number of kudu in the park appeared to increase exponentially after the 1992 drought until 2013, except during 2001–2007. During August–September 2004, the Malilangwe anthrax outbreak killed most of the kudu there (Clegg et al. 2007) and this outbreak may have spread to kudu in the adjacent northern section of Gonarezhou, before disappearing with the start of the October rains. Support for this possibility is provided by the stratum‐level estimates of kudu density in the park: density during 2007 was greater than during 2001 in four of the six strata in the park, but less in the northern‐most stratum, next to Malilangwe, and in one of the adjacent strata (Dunham 2002; Dunham et al. 2007). Carcasses found during the early stages of the Malilangwe outbreak suggested that female kudus were more susceptible than males. If the outbreak did spread amongst kudu in the north of Gonarezhou and if female kudu there were also more susceptible than males, this might explain the reduced rate of population increase during 2007–2013, cf. 1993–2001.

Impala was the only studied species that did not increase in number immediately after the 1992 drought. One might speculate that the absence of an increase was due to ration hunting of impala by park staff, with the annual offtake and annual recruitment being approximately equal. Ration hunting in the park ended soon after the start of FZS assistance to park management during late 2007, which may account for the rapid increase in impala estimates during 2007–2013. The impala was relatively little affected by the 2004 anthrax outbreak in Malilangwe (Clegg et al. 2007) and, if that outbreak did spread to Gonarezhou, it is likely that the impact on the Gonarezhou impala population was also slight.

An earlier assessment of the rate of increase of the Gonarezhou buffalo population immediately after the 1992 drought was unusually high (Dunham 2012). It is now known that significant numbers of buffalo were released in the park during 1998–2000 (Cumming 2016). These releases would have increased the apparent rate of population increase and so the current study considered buffalo estimates only for surveys after 2000.

The 2012–2013 translocations of large herbivores to Gonarezhou were of species that were already numerous in the park, or increasing in number, or both, prior to those releases (Dunham 2012). The translocations were a response to perceived management problems at the source sites and were of little or no conservation value for Gonarezhou. The aerial surveys show that the estimate of wildebeest number in the park peaked during 2013, just after the releases during August 2013, and then declined. In neighbouring Malilangwe, the number of wildebeest recorded killed by lions increased after the release of wildebeest there during 2008 and increased again after the release of additional wildebeest during 2009 (Clegg 2013). For Gonarezhou, there are no records of predator diets during this period, but the Malilangwe experience suggests that the wildebeest release in Gonarezhou may have prompted prey switching, perhaps by lions, so that wildebeest formed an increased proportion of the diet. A high susceptibility of the released wildebeest to predation may have been related to this water‐dependent species being translocated during the late dry season, when the released animals would have been dependent on limited sources of surface water for drinking. Lion kills in Hwange during the dry season were commonly close to water sources (Valeix et al. 2009). Elands were released in Gonarezhou at the same time as the wildebeest, but the eland is larger and less water‐dependent (Hempson et al. 2015) and thus, during the immediate post‐release period, the released elands were likely to have been more spatially dispersed than the wildebeests.

### 4.3 Possible Causes of Differing Population Trends After 2013

After 2013, the numbers of kudu, nyala, duiker, steenbok, impala, wildebeest, warthog, ostrich and possibly waterbuck declined in Gonarezhou, but the numbers of the larger herbivores (zebra, eland, buffalo, giraffe) did not. Possible reasons for these differing trends are now considered.

#### 4.3.1 Illegal Hunting

As part of the programme to reintroduce the black rhinoceros to Gonarezhou, antipoaching efforts in the park were increased and then maintained at a high level: patrol man‐days per annum increased 10‐fold after 2014, numbering ~50,000 during 2023 (R. Magwiro, GCT, pers. comm., 23 April 2024). Thus, it is unlikely that illegal hunting played any significant role in causing the observed population declines, which occurred during the same period.

#### 4.3.2 Rainfall

Dry season rainfall over Gonarezhou declined during this study, which may have reduced the availability of green grass for grazers at a time when food was in short supply (Mduma et al. 1999). Woody plants are impacted less than grasses by variation in rainfall (Walker et al. 1987). The 17 dry seasons of below‐average rainfall since 2004, especially if in combination with high grazing pressure (as is likely given that the abundance of the grazer species was increasing), may have prompted changes in the species composition of the grass layer, although no data on grass species composition are available. But if long‐term, rainfall‐induced variation in the food supply for herbivores was a factor in the declines observed in Gonarezhou, it was unlikely to be the only factor. That both the declining and non‐declining species included browsers, grazers and mixed feeders suggests that even if competition for food occurred, perhaps between species that declined and ones that did not, such competition did not cause all the declines.

In Hwange, census data collected during the late dry season at pans dependent on pumped water provided an explanation for population trends for large herbivore species (Valeix et al. 2008). Population trends were compared between three successive periods with different combinations of dry season rainfall and elephant abundance. The results suggested that dry season rainfall played an important role in herbivore population dynamics. But because most of the declines of herbivore populations occurred simultaneously with an increase in the elephant population, and a second increase in most populations occurred at a reduced rate compared with the first increase, the results also suggested that high densities of elephant had a negative impact on other herbivores in Hwange. Possibly there was competition for food during the dry season between elephants and the other herbivore species in key resource areas near waterholes; and possibly elephant‐induced vegetation changes modified the vulnerability of small to medium sized herbivores to predators (Valeix et al. 2008).

#### 4.3.3 Predation

Predator numbers in Gonarezhou increased during the period when the numbers of large herbivores were increasing prior to 2013, peaked soon afterwards and then declined as the numbers of smaller herbivores decreased. This is the expected pattern when predator numbers are regulated by their food supply, namely the numbers of herbivore prey (Hayward et al. 2007), many of which declined in Gonarezhou after 2013.

There is a strong similarity between the ~150 kg threshold between species regulated by predation and those regulated by food, suggested for Serengeti (Sinclair et al. 2003), and the threshold (between 136 and 200 kg) observed in Gonarezhou for large herbivore species that declined in number after 2013 and those species that did not. Also, the two herbivore species that started to decline first in Gonarezhou (steenbok and duiker) were the smallest of the studied species and thus would be the species susceptible to the greatest number of predator species. These observations suggest that predation likely played an important role in causing the declines observed in Gonarezhou. Steenbok and duiker estimates started to decline as the estimated number of impala increased after 2007. The impala is a preferred prey species of cheetah, wild dog and leopard (Hayward et al. 2007) and a principal prey elsewhere in south‐eastern Zimbabwe (Mbizah et al. 2012). The increase in impala abundance in Gonarezhou after 2007 coincided with increases in the numbers of these predators, and thus in predation pressure on other antelope species, such as duiker and steenbok.

The larger species of herbivore in Gonarezhou (buffalo, eland, giraffe and zebra) are susceptible to predation, but generally only by lion and, in the case of zebra, spotted hyaena. Estimates from camera surveys suggest that the density of lion (but not leopard) in Gonarezhou is relatively low (Loveridge et al. 2022, 2024), which may indicate that the larger herbivore species are subject to less predation in Gonarezhou than in other southern African ecosystems. The low density of lion in Gonarezhou may in part be a consequence of excessive numbers of lion being killed in areas around the park (Groom et al. 2014), particularly on the Mozambican game farms along the eastern boundary of the park. There is no density estimate for spotted hyaena in Gonarezhou, but many of those photographed carried snares or had snare wounds, which might suggest that the hyaena population is subject to significant anthropogenic mortality, and possibly kept below carrying capacity by such mortality. Despite its relatively large body size, the zebra is often thought to be regulated by predation and not food limitation (Duncan et al. 2024), but currently there is no evidence of this in Gonarezhou.

While it is likely that the population declines observed in Gonarezhou were caused by predation, it is less obvious why the population trends of the smaller herbivore species changed so abruptly, from increasing before 2013 to decreasing afterwards.

#### 4.3.4 Elephant Impacts on Vegetation

Concerns about the impact of elephant on the vegetation in Gonarezhou were first noted during the 1960s (Sherry 1975) and have continued since (O'Connor, Ferguson, et al. 2024; O'Connor, Pallett, et al. 2024). While there have been various studies of the Gonarezhou vegetation since 1993 (e.g., Cunliffe et al. 2012; Gandiwa et al. 2012; Gandiwa, Magwati, et al. 2011; Foster et al. 2024; Gandiwa and Kativu 2009; Zisadza‐Gandiwa et al. 2013; Mandinyenya et al. 2025), none have monitored changes in vegetation structure or species composition in parallel with the aerial survey programme. Elephant impacts on vegetation may affect other herbivore species directly by increasing or decreasing their food supply; or indirectly by modifying the vegetation structure in ways that change the other herbivores' vulnerability to predation.

Elsewhere in southern Africa, spotted hyaenas and lions occasionally kill non‐adult elephants (Salnicki et al. 2001; Loveridge et al. 2006; Power and Compion 2009), but the population impact of non‐human predation on elephants is generally negligible. The noticeable decline in the rate of increase in the estimated number of elephants in female herds in Gonarezhou after 2014, and the decline of that rate to zero after 2018, likely reflects food limitation. A food shortage would be expected to lead to intraspecific competition for food, a decline in the condition of at least some individual elephants, and likely reduced fecundity or increased juvenile mortality (Foley et al. 2008; Lee et al. 2011). Probably, interspecific competition for food also occurred. In Sengwa, Zimbabwe, 82% of elephant mouthfuls of browse were taken from < 2 m above the ground, with 60% taken from < 1.2 m (Guy 1976). Thus, in terms of feeding height, elephants likely compete for food with other species of large browser and mixed feeder. There is also likely to be competition at the level of plant species because the elephant diet is wide ranging: elephants in Sengwa ate at least 133 plant species (Guy 1976).

Forbs (non‐woody, dicotyledonous plants) are often an important component of the diet of elephants and other mixed feeder and browser species, including impala, kudu and eland (Dunham 1980; Owen‐Smith and Cooper 1989; Watson and Owen‐Smith 2000; Clegg 2008). In Gonarezhou, forbs tend to be more abundant in riverine vegetation communities (Cunliffe et al. 2012). Elephants eat forbs by removing the entire plant (Clegg and O'Connor 2025) and, in Kenyan rangeland experiments, the presence of elephants reduced forb cover by half (Kimuyu et al. 2017). High densities of elephants, as in Gonarezhou, would likely have an even greater impact on the availability of forbs as food for other large herbivores.

As O'Connor, Ferguson, et al. (2024) point out, where the geographic distributions of elephant and *Colophospermum mopane* overlap in southern Africa, *C. mopane* forms a major proportion of the elephant diet (e.g., Jarman 1971; Williamson 1975; Guy 1976; Kos et al. 2012). But in those areas, *C. mopane* forms only a small proportion of the diet of smaller species of mixed feeder or browser, for example, impala, kudu (Jarman 1971; Conybeare 1975; Dunham 1980; Kos et al. 2012). *Colophospermum mopane* trees and shrubs coppice readily after being fed on by elephants. In Gonarezhou, the conversion of tall mopane woodland to a mopane bushland during 8 years of use by a high density of elephants did not decrease the canopy volume below 3 m height, nor the overall canopy volume (O'Connor, Ferguson, et al. 2024). But mopane woodland impacted by elephant was characterised by a shrub layer with a changed composition and fewer woody species (O'Connor, Pallett, et al. 2024). In riverine woodland on the south bank of the Runde river, the cover of mature trees declined by 26% between the 1970s and 2024; and the total woody cover declined by 59% between 2013 and 2016, and by a further 23% by 2018 (O'Connor et al. 2026). Mean woody species richness declined across the park during 2010–2023 from 26 to 20 per plot (Mandinyenya et al. 2025). The decline in species richness indicates that, for an average of six species per plot, their biomass in the plot declined to zero.

Elephant dietary preferences for woody plant species may change when a high density of elephants persists, as in Hwange where plant species initially avoided (selected against) were later eaten in proportion to their abundance in the environment (Ferry et al. 2021). In other words, when at a persistent high density, elephants became less fussy about which woody species they ate. Taken all together, these observations suggest that even when the density of elephants is high, elephant impacts on *C. mopane* shrubs have a small or negligible effect on the diets of smaller mixed feeders or browsers. But elephant impacts on other shrub species—which form the bulk of the diets of these smaller mixed feeders and browsers—are likely to reduce not only the species richness but also the biomass of other shrubs, and hence the food supply for other species of smaller mixed feeders and browsers.

The largest species of browser in Gonarezhou is the black rhinoceros, which was recently reintroduced to the park. As with the elephant, its young are occasionally killed by predators (Plotz and Linklater 2009), but adults are mainly immune to non‐human predation. A high degree of spatial, dietary and feeding height overlap between black rhinoceros and elephant provided strong evidence that the two species compete for food in Gonarezhou (van der Westhuizen et al. 2025). *Colophospermum mopane* contributed the second highest proportion of browse for rhinoceroses, although elephants and rhinoceroses were segregated in the diameter of twigs taken. Elephants often removed whole branches, leaving behind low‐quality browse on stems whose diameters are too large for black rhinoceroses (or other large herbivores, e.g., kudus) to feed upon. But elephants apparently increased the future availability of mopane browse for rhinoceroses by causing shrubs to coppice, resulting in twigs too small for elephants, but of a suitable diameter, and at an appropriate height, for rhinoceroses. Because *C. mopane* forms a substantial proportion of the rhinoceros diet in Gonarezhou (even though, as van der Westhuizen et al. (2025) pointed out, it is considered of low palatability for black rhinoceros), the black rhinoceros may be less impacted by competition for food with elephant than the other species of browser or mixed feeder.

For ~20 years after the 1992 drought, the estimated number of elephants in Gonarezhou increased at 6% per annum. The mean density of all elephants exceeded 2 km^−2^ during 2013 and reached 2.25 km^−2^ the following year, the latter density a 2.7‐fold increase since 1995. For elephants in female herds, the rate of increase was 6.6% per annum. Much of Gonarezhou is > 15 km from a perennial river and elephants concentrate in the vicinity of these rivers during the dry season and so density here is greater than the park mean density. The survey estimates for elephants in female herds suggest that after 2014 (when their mean density reached 2.0 km^−2^), there was an abrupt reduction in their rate of increase, which was essentially zero after 2018 (Figure 5b). The declines in the herbivore populations occurred during the period, 2014 and onwards, when the estimated mean density of elephants in female herds had levelled off at ~2 km^−2^, likely as a result of food limitation for the elephants in female herds within the vicinity of the perennial rivers. Meanwhile, the number of elephants in bull groups continued to increase at 6.4% per annum. Elephant bulls, being larger, could range and forage further from sources of drinking water than female herds containing youngsters (Stokke and du Toit 2002). Female herds in Hwange were usually 2–6 km from water during the dry season and bulls usually 4–10 km, while the maximum distance from water for both sexes was ~15 km (Conybeare 1991).

Elephant density in Gonarezhou during 2014 was greater than at any time since at least 1980 (Dunham 2012, 2025a) and probably greater than at any time during the century before then, because there were < 5000 elephants in all Zimbabwe at the start of the twentieth century (Cumming et al. 1997). Elephant impacts on the Gonarezhou woody vegetation have been a concern for more than five decades (Sherry 1975) and since 2014 the impacts have been extremely severe, with reductions in the biomass of woody species other than *C. mopane* . These impacts would have caused both intra‐specific competition for food and interspecific competition for food with browser and mixed feeder species. For these other herbivore species, competition for food would likely have caused poor body condition and an increased vulnerability to predation (Pole et al. 2004).

The impact of elephant, that transformed *C. mopane* woodlands to bushlands in Gonarezhou and reduced or removed other woody species, was expected to have a negative effect on other mixed feeder or browser species, ranging from black rhinoceros and giraffe to duiker and steenbok, because these species rely on a variety of plant species in their diets (O'Connor, Pallett, et al. 2024). The current study has revealed that there have already been declines in the abundance of some large herbivores in Gonarezhou, but the situation is not straightforward. While food shortages caused by elephant impacts on the vegetation may have been the ultimate cause of the declines in the numbers of the smaller mixed feeder and browser species, predation was likely the proximate cause. Predation likely also caused the declines of the smaller grazer species. These probably became more vulnerable to predation as their food supply at a critical time of year was reduced by the decrease in dry season rainfall; and likely also because of prey switching by predators as the numbers of other prey species declined. The larger species (giraffe, buffalo, eland, zebra) have not declined in abundance. Giraffe (a browser) and eland (predominantly a browser) are less dependent on drinking water and, because of their size, they are both less susceptible to predation, except by lion, and can range further from the perennial rivers (and the most severely elephant‐impacted vegetation) during the dry season. The supply of grass that buffalo and zebra eat was unlikely to be impacted by elephant and, again because of their size, they are less susceptible to predation than smaller species. Furthermore, the non‐ruminant zebra and very large buffalo can survive on a grass diet that is poorer in quality than the smaller grazing herbivore species (Bell 1971; Jarman 1974; Sinclair 1974).

Elephant conservation in Gonarezhou might be seen as a success, because the number of elephants there has increased. In contrast, elephant numbers have declined during the same period in two of the other three major subpopulations in Zimbabwe (Dunham 2015). But the negative impacts on biodiversity of the increased number of elephants in Gonarezhou were highlighted recently as a management concern (O'Connor 2025). This study suggests that the current high density of elephants in Gonarezhou may have had knock‐on and adverse effects on the populations of some large herbivore species. The density of carnivores is positively related to the abundance of their prey (Hayward et al. 2007), and so it is unsurprising that there appear to have been further knock‐on effects of the high elephant density on the predator populations in Gonarezhou, which have declined (Groom and Watermeyer 2017; Shito and Watermeyer 2024) as the populations of the smaller herbivores (their prey) have declined.

## Author Contributions

**Kevin M. Dunham:** formal analysis (lead), investigation (lead), methodology (lead), visualization (lead), writing – original draft (lead), writing – review and editing (lead).

## Funding

The author has nothing to report.

## Conflicts of Interest

Since 2001, K.M.D. has often been contracted by WWF, FZS, or GCT to lead Gonarezhou survey teams.

## Data Availability

The data that support the findings of this study were derived from the survey reports (Section 2.2) which are publicly available.
